# Matrix-M™ Adjuvant Induces Local Recruitment, Activation and Maturation of Central Immune Cells in Absence of Antigen

**DOI:** 10.1371/journal.pone.0041451

**Published:** 2012-07-23

**Authors:** Jenny M. Reimer, Karin H. Karlsson, Karin Lövgren-Bengtsson, Sofia E. Magnusson, Alexis Fuentes, Linda Stertman

**Affiliations:** Isconova AB, Uppsala, Sweden; Institut National de la Santé et de la Recherche Médicale U 872, France

## Abstract

Saponin-based adjuvants are widely used to enhance humoral and cellular immune responses towards vaccine antigens, although it is not yet completely known how they mediate their stimulatory effects. The aim of this study was to elucidate the mechanism of action of adjuvant Matrix-M™ without antigen and Alum was used as reference adjuvant. Adjuvant Matrix-M™ is comprised of 40 nm nanoparticles composed of *Quillaja saponins*, cholesterol and phospholipid. BALB/c mice were subcutaneously injected once with, 3, 12 or 30 µg of Matrix-M™, resulting in recruitment of leukocytes to draining lymph nodes (dLNs) and spleen 48 h post treatment. Flow cytometry analysis identified CD11b^+^ Gr-1^high^ granulocytes as the cell population increasing most in dLNs and spleen. Additionally, dendritic cells, F4/80^int^ cells, T-, B- and NK-cells were recruited to dLNs and in spleen the number of F4/80^int^ cells, and to some extent, B cells and dendritic cells, increased. Elevated levels of early activation marker CD69 were detected on T-, B- and NK-cells, CD11b^+^ Gr-1^high^ cells, F4/80^int^ cells and dendritic cells in dLNs. In spleen CD69 was mainly up-regulated on NK cells. B cells and dendritic cells in dLNs and spleen showed an increased expression of the co-stimulatory molecule CD86 and dendritic cells in dLNs expressed elevated levels of MHC class II. The high-dose (30 µg) of Matrix-M™ induced detectable serum levels of IL-6 and MIP-1β 4 h post administration, most likely representing spillover of locally produced cytokines. A lesser increase of IL-6 in serum after administration of 12 µg Matrix-M™ was also observed. In conclusion, early immunostimulatory properties were demonstrated by Matrix-M™ alone, as therapeutic doses resulted in a local transient immune response with recruitment and activation of central immune cells to dLNs. These effects may play a role in enhancing uptake and presentation of vaccine antigens to elicit a competent immune response.

## Introduction

Adjuvants are compounds added to vaccine antigens to facilitate and enhance activation of the innate and adaptive immune responses, to improve the immunogenicity and efficacy of vaccines. Adjuvants exert their effects through various mechanisms, but also the nature and identity of the antigen(s) contributes to the immune response [1]. The accumulated knowledge on adjuvant mode of action is primarily based on data from studies of adjuvants formulated with vaccine antigens. However, to gain unbiased information on the specific immune stimulation elicited by the adjuvant, studies without antigen(s) are required. Basic research on adjuvant properties is central in order to better understand how adjuvants and antigens work in concert, generating a safe and efficacious immune response.

Saponins, particularly those obtained from *Quillaja saponaria* Molina, are known potent adjuvants and *Quillaja saponins* (QS) have for long been used in animal vaccines. Saponin-based adjuvants can be formulated in different ways; in free form [2], with aluminium hydroxide [3], in ISCOMs (immunostimulating complex) [4] or in ISCOM-Matrix/Matrix structures [5]. QS constitute a heterogeneous mixture of related but different chemical structures with various immunostimulatory activities, safety profiles and particle forming properties. By purification of the QS raw material, distinctive fractions with different characteristics can be defined.

The ISCOM, a potent adjuvant formulation first described in 1984 by Morein and co-workers [4], consist of stable complexes composed of saponin, cholesterol, phospholipid and incorporated antigen(s). The hallmarks of the ISCOM technology are the dose-sparing potential [6], induction of high and long-lasting antibody titers and potent T cell responses [7]. However, later it was shown that antigen incorporation is not critical for these immune properties. Antigen and empty ISCOMs *i.e.* ISCOM-Matrix/Matrix could simply be mixed with sustained vaccine efficacy [5]. In this study we use a novel adjuvant formulation based on two different Matrix particles made from two separate purified fractions of saponins, yielding Matrix-A™ and Matrix-C™ [8]. These Matrix particles, approximately 40 nm large, are subsequently mixed at defined ratios to get the Matrix-M™ adjuvant.

Formulated saponin-based adjuvants are thought to enhance cell trafficking and activation of immune cells, *i.e.* components responsible for inducing cytokine production and facilitating antigen uptake, processing and presentation on MHC class I and II [9]. Moreover, convincing data suggests that co-delivery of the saponin-based adjuvant ISCOMATRIX™ and antigen is central for CD8^+^ T cell induction and that their simultaneous drainage to a common lymph node is crucial for the adjuvant effect in general [10]. Interestingly though, unpublished data show that premixing of Matrix-M™ and influenza antigen is not required to mount a potent humoral immune response. Matrix-M™ and antigen could be administered up to 24 h apart with sustained IgG1 and IgG2a antibody responses.

Currently, the ISCOM-Matrix/Matrix saponin-based adjuvants Matrix-M™ [11] and ISCOMATRIX™ [12] have entered and moved forward into human clinical trials. Other ISCOM-Matrix/Matrix adjuvants are available in commercial veterinary vaccines *e.g.* Matrix-C™ [13]. The assembled efficacy data on these and other saponin-formulated vaccines is convincing, however, their mode of action still needs to be further investigated. In this study we have chosen to elucidate the early immunostimulatory properties of Matrix-M™ in mice without influence of antigen, in order to further characterize its mode of action. Accordingly, understanding the inherent, antigen-independent properties of adjuvants is thus essential for the development of safe and effective vaccines.

## Materials and Methods

### Adjuvant preparation

Matrix-M™ (AbISCO®-100, Isconova AB, Uppsala, Sweden), a mixture of Matrix-A™ and –C™ at the ratio of 85∶15, was used. Matrix-A™ and –C™ were prepared from separately purified fractions of QS subsequently formulated with cholesterol and phospholipid into Matrix particles. The Alum used in this study was 2% Alhydrogel (Al(OH)_3_, Brenntag Biosector, Frederikssund, Denmark).

### Animals and experimental design

Eight weeks old female BALB/c mice were purchased from Scanbur (Stockholm, Sweden) and kept at the National Veterinary Institute (SVA, Uppsala, Sweden). All animal experiments were approved by Uppsala Ethical Committee (Permit numbers: C 50/7, C 191/11). The animals were kept in accordance with national guidelines. Mice were injected subcutaneously (s.c.) at the base of the tail with 100 µl Matrix-M™. The therapeutic dose range of Matrix-M™ in mice is 3–12 µg, thus 3 and 12 µg were evaluated in this study. Moreover, mice were injected with a high-dose of Matrix-M™ (30 µg). Control mice received 100 µl PBS, pH 7.4. In a separate experiment mice were injected s.c. at the base of the tail with 100 µl Alum (1%) corresponding to a 500 µg Alum dose and naïve mice were used as negative controls. Matrix-M™-treated mice were included as controls. Draining lymph nodes (dLNs, inguinal), spleen and blood were collected 4, 24 and 48 h after injection, except for 12 µg Matrix-M™ where dLN and spleen were collected at 24 and 48 h. Blood was drawn from the tail vein at all time points.

### Quantification of cytokines

The presence of cytokines and chemokines in serum was measured using Cytometric Bead Array (CBA, BD Biosciences, Erembodegem, Belgium) according to the manufacturer's instructions. The protein flex sets used were IL-1β, IL-2, IL-4, IL-6, IL-10, IL-12p70, MIP-1β, IFN-γ, TNF, RANTES and GM-CSF. Serum samples were diluted 1∶4 with assay diluent buffer. Fluorescence measurement was performed using a FACSCanto flow cytometer (BD Biosciences) and data were analyzed using the FCAP Array software (v. 1.0.1, BD Biosciences).

### Cell preparation

Spleen and dLNs were collected in cold PBS and processed to single cell suspensions by passage through a 23G needle. Cells from the two dLNs were pooled. Splenocytes were incubated in ammonium chloride for 5 min, washed with PBS containing 2% FBS and passed through a 100 µm cell strainer. Cells were resuspended in staining buffer (PBS, pH 7.4, 0.5% BSA, 2 mM EDTA, 0.1% sodium azide). The viable cell concentration was determined by staining with Trypan blue and phase contrast microscopy.

### Morphological analysis

Single cell suspensions were centrifuged onto microscope slides (10^5^ cells/slide, 500 rpm, 5 min) using a cytocentrifuge (Shandon, Cheshire, United Kingdom). Slides were air-dried at RT, stained with May-Grünwald-Giemsa (Merck, Darmstadt, Germany) and analyzed using a Leica CTRMIC microscope (Leica Microsystems, Wetzlar, Germany). The analysis of the slides was done in an unblinded fashion.

### Flow cytometry analysis

Cell suspensions were prepared as described above and incubated 20 min at 4°C with anti-mouse CD16/CD32 antibody (Mouse BD Fc Block™). Cells were then transferred to a 96-well microtiter plate and incubated with antibodies for 30 min at 4°C (5×10^5^ cells, 100 µl/well). Antibodies used were anti-mouse; CD3:APC (145-2C11), CD4:PE-Cy7 (RM4-5), CD8:APC-H7 (53-6.7), CD49b:FITC (DX5), CD69:PE (H1.2F3), CD86:PE (GL1), F4/80:Alexa 647 (Cl:A3-1, AbD Serotec), Gr-1:PE-Cy7 (RB6-8C5), CD11b:APC-Cy7 (M1/70), CD11c:APC (HL3), CD19:PE-Cy7 (1D3) and I-A/I-E:FITC (MHC class II, 2G9). CD86 and MHC class II expression were only analyzed on B cells and dendritic cells (DCs). Appropriate fluorochrome-conjugated isotype antibodies were used as controls. Cells were washed and incubated 10 min at RT with 7-AAD to stain non-viable cells. All staining procedures were conducted on ice and reagents were purchased from BD Biosciences unless otherwise stated. Cells were analyzed using a FACSCanto flow cytometer (BD Biosciences), 50 000 events per sample were collected. Retrieved data was analyzed using the FACSDiva software (v. 5.0.2, BD Biosciences).

### Statistical analysis

Data were analyzed using the non-parametric tests Mann-Whitney or Kruskal-Wallis test with Dunn's post test (GraphPad Prism 5.01 for Windows, GraphPad Software, San Diego, California, USA). Statistical significance was assigned at a p value of <0.05.

## Results

### High-dose Matrix-M™ increases IL-6 and MIP-1β levels in serum

**Figure 1:**
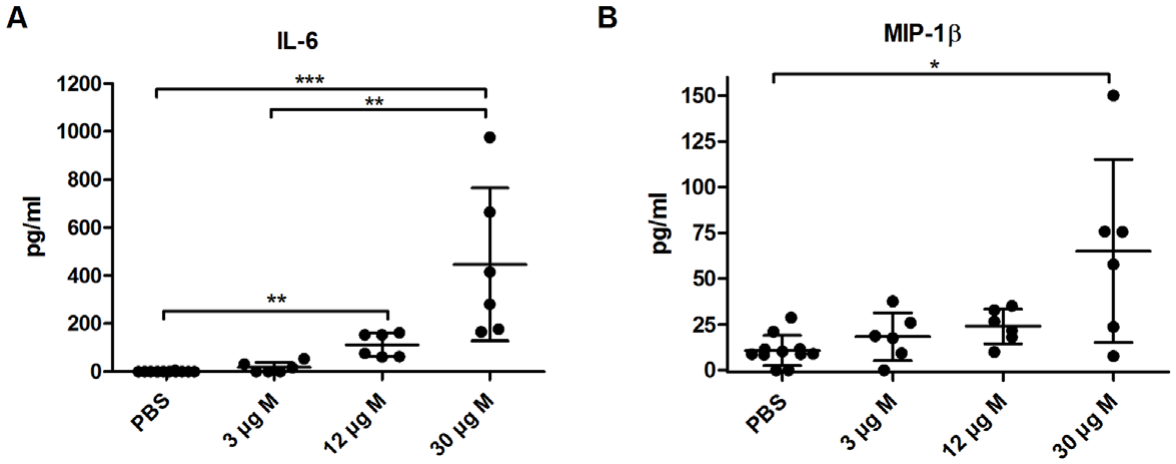
High-dose Matrix-M™ increases the IL-6 (A) and MIP-1β (B) levels in serum. Mice were subcutaneously injected at the base of the tail with 3, 12 or 30 µg Matrix-M™ or PBS. Cytokine levels in serum were detected by Cytometric Bead Array (CBA) 4 h after injection. Data is presented as mean ± SD (n = 6–8 mice). Significant differences using Kruskal-Wallis test with Dunn's posttest are outlined with *, *p<0.05*; **, *p<0.01*; ***, *p<0.001*. M, Matrix-M™.

Mice were injected s.c. with 3, 12 or 30 µg of Matrix-M™ or Alum and the cytokine/chemokine levels in serum were analyzed using CBA after 4 and 24 h. Thirty µg of Matrix-M™ significantly increased serum levels of IL-6 (446±319 pg/ml, p<0.001) and to a lesser degree also MIP-1β (65±50 pg/ml, p<0.05) (Figure 1). The IL-6 level was also increased in 12 µg Matrix-M™-treated mice compared to PBS control (110±49 versus 0.38±1.3, p<0.01). IL-6 and MIP-1β levels returned to near background levels at 24 h (data not shown). Increased levels of circulating IL-6 and MIP-1β were not observed with 3 µg Matrix-M™ or Alum (Figure 1 and data not shown). The levels of IL-1β, IL-2, IL-4, IL-10, IL-12p70, IFN-γ, TNF and GM-CSF were below detection levels and the levels of RANTES retrieved after administration of Matrix-M™ or Alum did not differ from the levels in the control mice (data not shown).

### Matrix-M™ induces enhanced cell numbers in draining lymph nodes and spleen

**Figure 2:**
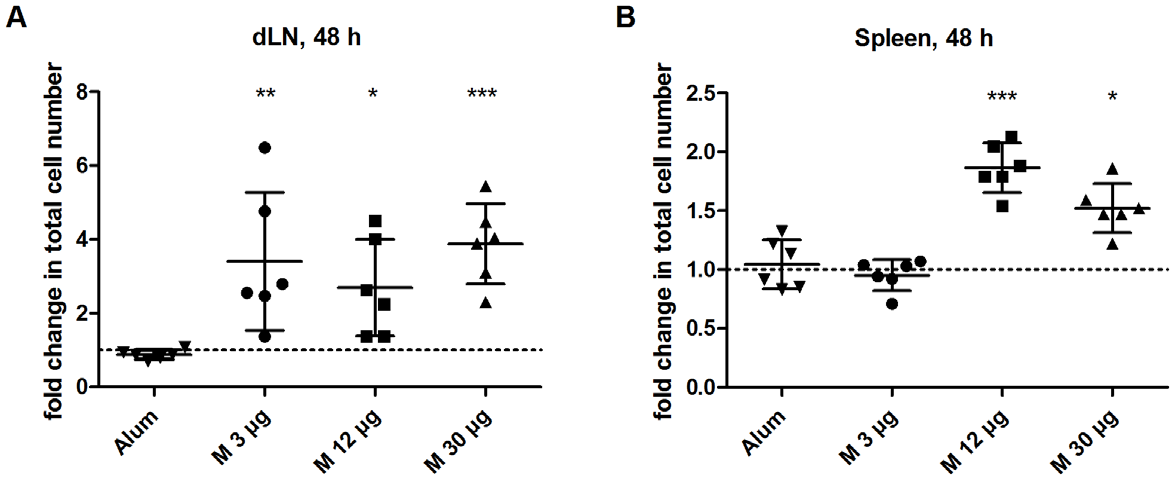
Fold change in total cell numbers in dLNs and spleen after Matrix-M™ or Alum treatment. Alum or 3, 12 or 30 µg Matrix-M™ were injected subcutaneously at the base of the tail. After 48 h cells from the two draining lymph nodes (dLNs) (A) and the spleen (B) were prepared and total cell counts were performed. The relative difference in cell numbers between 3, 12 or 30 µg Matrix-M™- and PBS-treated mice or Alum-treated and naïve mice, respectively are presented. Dotted line represent no difference in cell number between treated and control mice (fold change = 1). Data is shown as mean ± SD (n = 6–8 mice). Significant differences between Matrix-M™ - and PBS-treated mice using Kruskal-Wallis test with Dunn's posttest or between Alum-treated and naïve mice using Mann-Whitney test are outlined with *, *p<0.05*; **, *p<0.01*; ***, *p<0.001*. M, Matrix-M™.

Following s.c. injection with 3, 12 or 30 µg Matrix-M™ or Alum, single-cell suspensions prepared from spleen and dLNs were analyzed after 4, 24 and 48 h. Total spleen and lymph node cell counts showed that Matrix-M™ increased the total cell number compared to the PBS control. For the time points and Matrix-M™ doses analyzed, the highest number of cells was observed at 48 h post injection in dLNs. The total cell number increased three-fold compared to PBS treatment, independent of Matrix-M™ dose tested (p<0.01 (3 µg), p<0.05 (12 µg) and p<0.001 (30 µg), Figure 2). In spleen, the highest number of cells was detected already at 24 h (data not shown) and remained at 48 h post injection (Figure 2). The increase in splenocyte numbers were observed for the higher doses tested. Three µg Matrix-M™ did not significantly increase the cell number compared to the control (0.95±0.13 versus 1.0±0.1, p = ns), whereas 12 µg (1.9±0.2, p<0.001) and 30 µg (1.5±0.2, p<0.05) Matrix-M™ almost doubled the number of splenocytes at 48 h. However, Alum treatment did not result in a detectable increase in total cell numbers in dLNs or spleen at 4, 24 and 48 h (Figure 2 and data not shown).

**Figure 3:**
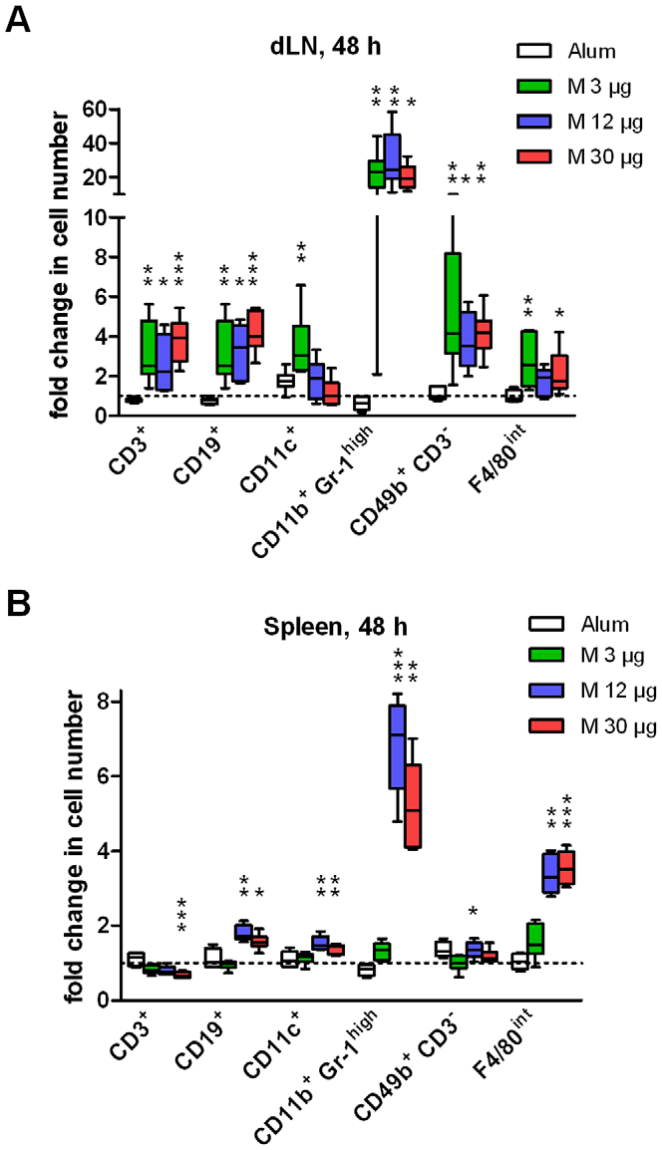
Fold change in cell populations in dLNs and spleen after Matrix-M™ or Alum treatment. Alum or 3, 12 or 30 µg Matrix-M™ were injected subcutaneously at the base of the tail. After 48 h cells from the draining lymph nodes (dLNs) (A) and the spleen (B) were prepared and analyzed by flow cytometry. The relative difference in cell numbers between 3, 12 or 30 µg Matrix-M™- and PBS-treated mice or Alum-treated and naïve mice, respectively are presented. Dotted line represent no difference in cell number between treated and control mice (fold change = 1). Data is shown as median (n = 6–8 mice), whiskers show min and max value. Significant differences between Matrix-M™ - and PBS-treated mice using Kruskal-Wallis test with Dunn's posttest or between Alum-treated and naïve mice using Mann-Whitney test are outlined with *, *p<0.05*; **, *p<0.01*; ***, *p<0.001*. M, Matrix-M™.

Granulocytes identified as CD11b^+^ Gr-1^high^ was the population that increased the most in both dLN and spleen at 48 h in Matrix-M™-treated mice relative to PBS control (Figure 3). Mice treated with 12 or 30 µg Matrix-M™ had a seven- and five-fold increase of granulocytes, respectively (p<0.001 and p<0.05, respectively) in spleen and all doses of Matrix-M™ led to about 20 times more granulocytes in dLNs compared to PBS control (p<0.01 (3 and 12 µg) and p<0.05 (30 µg)). The large error bar on the lower side for 3 µg is due to one outlier (fold change 2.09). Although the granulocytes were the population with the highest relative increase in cell numbers, the percentage of granulocytes of total cell numbers was only 0.3% in dLNs and 7% in spleen (30 µg Matrix-M™). All doses of Matrix-M™ resulted in a two- to four-fold increase in T-, B- and NK-cells in dLNs (Figure 3). A significant increase of DCs was detected in 3 µg Matrix-M™-treated mice (p<0.01) and 3 and 30 µg Matrix-M™ significantly increased F4/80 intermediate cells, primarily macrophages (p<0.01 and p<0.05, respectively). In spleen, 12 and 30 µg doses of Matrix-M™ induced a small increase (less than two-fold) of B cells and dendritic cells and a three-fold increase of F4/80 intermediate cells. Twelve µg Matrix-M™ induced a slight but significant increase (1.4±0.2, p<0.05) of NK cells. However, the T-cell population remained unchanged except in the 30 µg Matrix-M™-treated mice, where the number of T cells decreased compared to the PBS control (0.7±0.1 versus 1.0±0.1, p<0.001, Figure 3). Alum treatment did not have an effect on the different cell populations analyzed (Figure 3).

### Kinetics and characterisation of recruited CD11b^+^ Gr-1^high^ granulocytes

**Figure 4:**
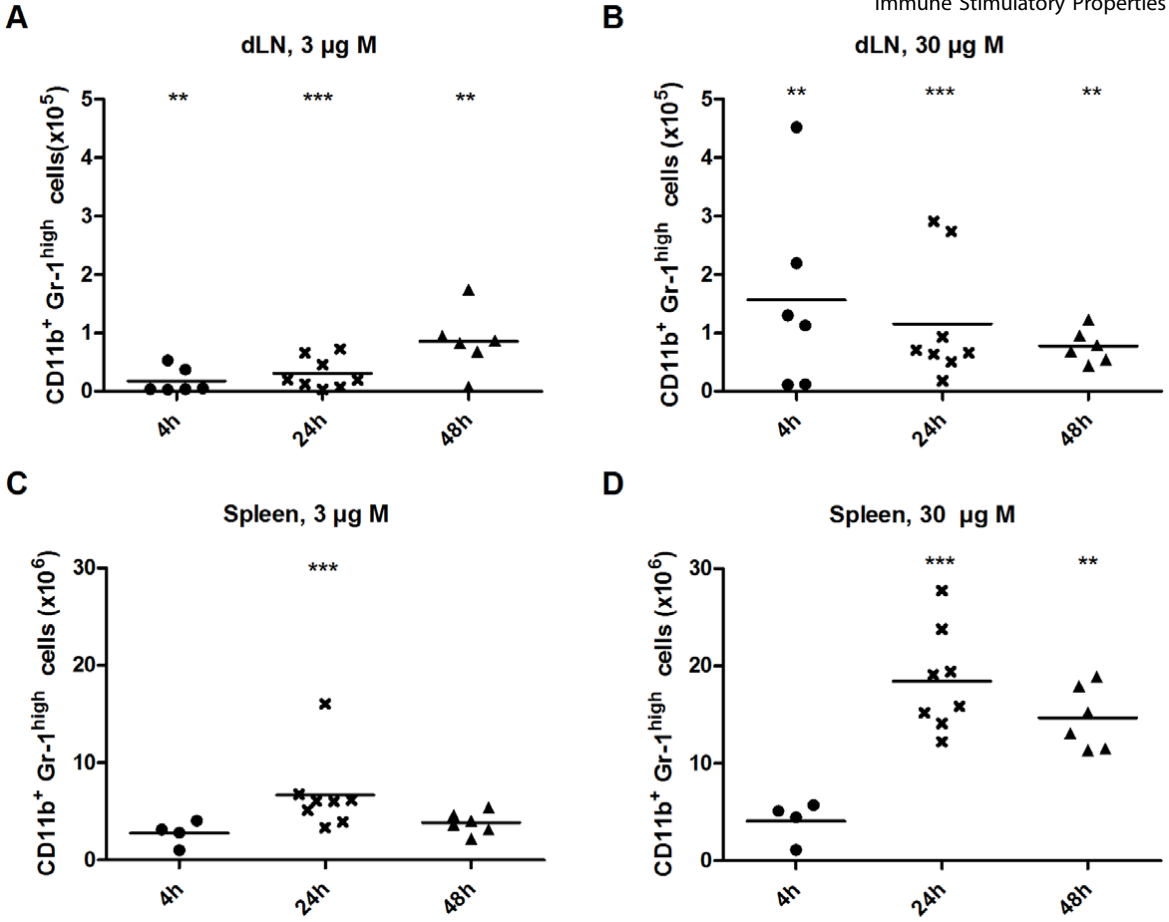
Total number of granulocytes (CD11b^+^ Gr-1^high^) in dLNs and spleen after Matrix-M™ treatment. 3 or 30 µg Matrix-M™ were injected subcutaneously at the base of the tail and after 4, 24 or 48 h cells were prepared and counted from the draining lymph nodes (dLNs) (A and B) and the spleen (C and D). The mean of 4–8 mice/group is shown. Significant differences from the PBS treatment using Mann-Whitney test are outlined with *, *p<0.05*; **, *p<0.01*; ***, *p<0.001*. M, Matrix-M™.

In dLNs of mice treated with 3 µg Matrix-M™ the CD11b^+^ Gr-1^high^ granulocyte population increased over time (Figure 4). However, the high dose of 30 µg Matrix-M™ yielded a maximum number of granulocytes detected in the dLNs already at 4 h, which then decreased over time, reaching the same granulocyte number as in mice treated with 3 µg Matrix-M™ at 48 h. The difference in kinetics elicited by the different Matrix-M™ doses was not detected in spleen (Figure 4), where the increase of granulocytes was observed 24 h post treatment with both 3 and 30 µg Matrix-M™.

**Figure 5:**
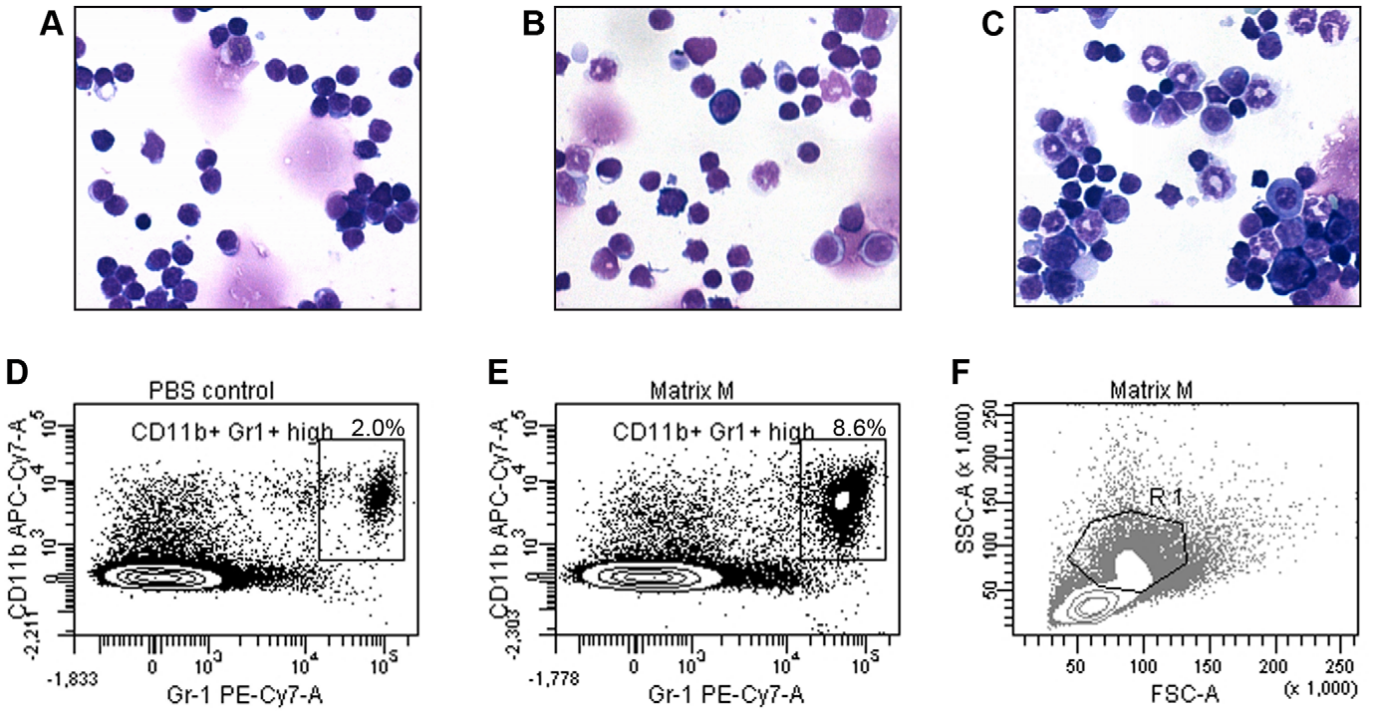
Characterization of splenocytes after Matrix-M™ treatment. Morphology of May-Grünwald-Giemsa stained splenocytes taken from a PBS-treated mouse (A), mice treated with 3 µg Matrix-M™ (B) or 30 µg Matrix-M™ (C) (original magnification X40). Representative dot plots of the CD11b^+^ Gr-1^high^ population in spleen at 48 h after injection of PBS (D) or 30 µg Matrix-M™ (E). More than 90% of the cells in the CD11b^+^ Gr-1^high^ gate can be found in the R1 region in FSC-SSC plot (F). Contour interval is 15%. M, Matrix-M™.

Cytospin preparations of splenocytes taken from mice 48 h post treatment with 3 or 30 µg Matrix-M™ displayed an increase in cells with neutrophil-like morphology *i.e.* segmented ring-shaped nucleus and granulated cytoplasm (Figure 5). These immature myeloid cells were not recovered in spleens of PBS-treated mice. Moreover, splenocyte preparations from mice 48 h post treatment with 30 µg Matrix-M™ revealed a prominent population of CD11b^+^ Gr-1^high^ cells compared to control (Figure 5).

### Matrix-M™ activates immune cells in dLNs and spleen

**Figure 6:**
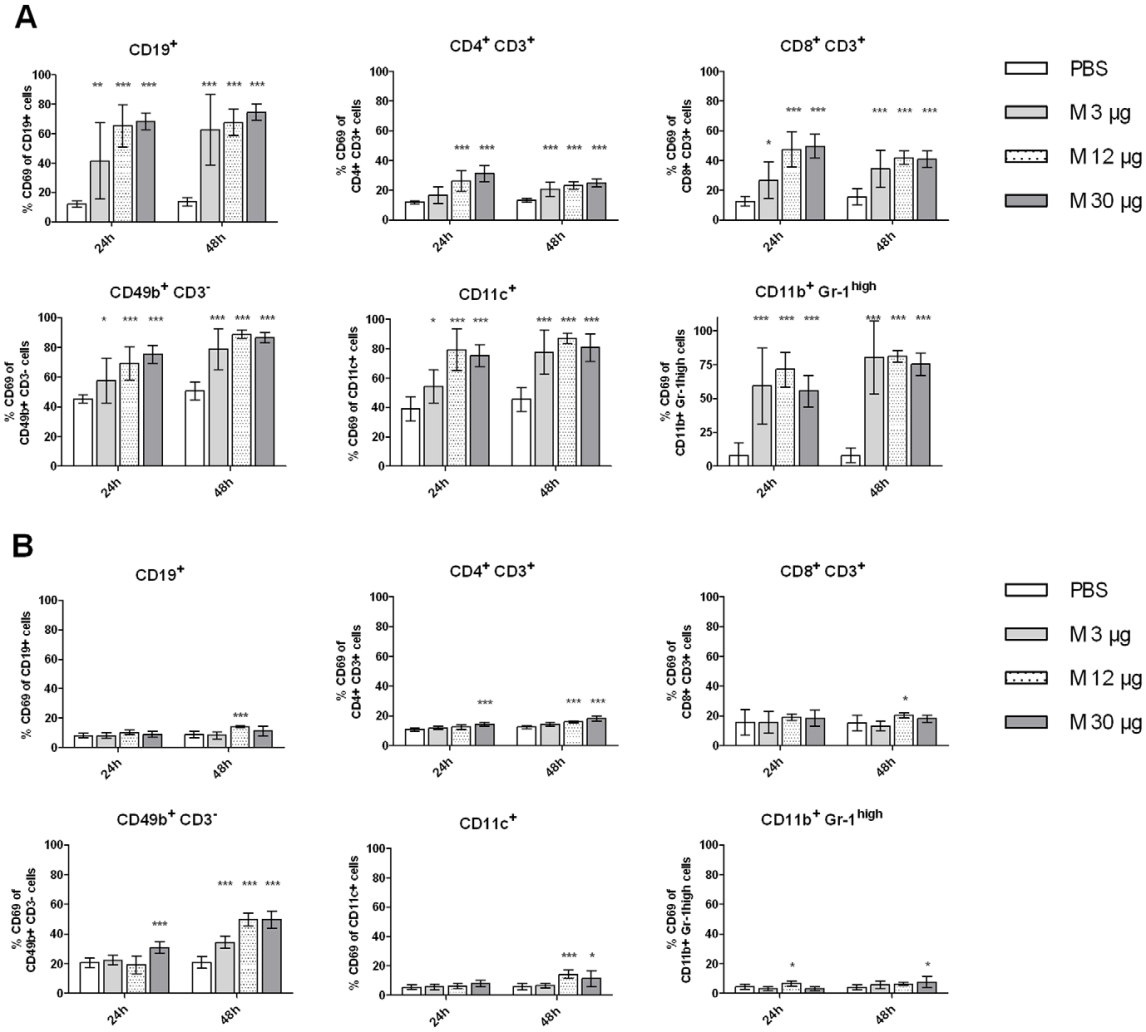
Increased expression of CD69 after Matrix-M™ treatment. 3, 12 or 30 µg Matrix-M™ or PBS were injected subcutaneously at the base of the tail and at 24 or 48 h post treatment cells were prepared and the expression of CD69 on cells from the draining lymph nodes (A) or the spleen (B) were analyzed. Data is presented as mean ± SD (n = 6–8 mice). Significant differences between Matrix-M™ and PBS treatments using Kruskal-Wallis test with Dunn's posttest are outlined with *, *p<0.05*; **, *p<0.01* ; ***, *p<0.001*. M, Matrix-M™.

**Figure 7:**
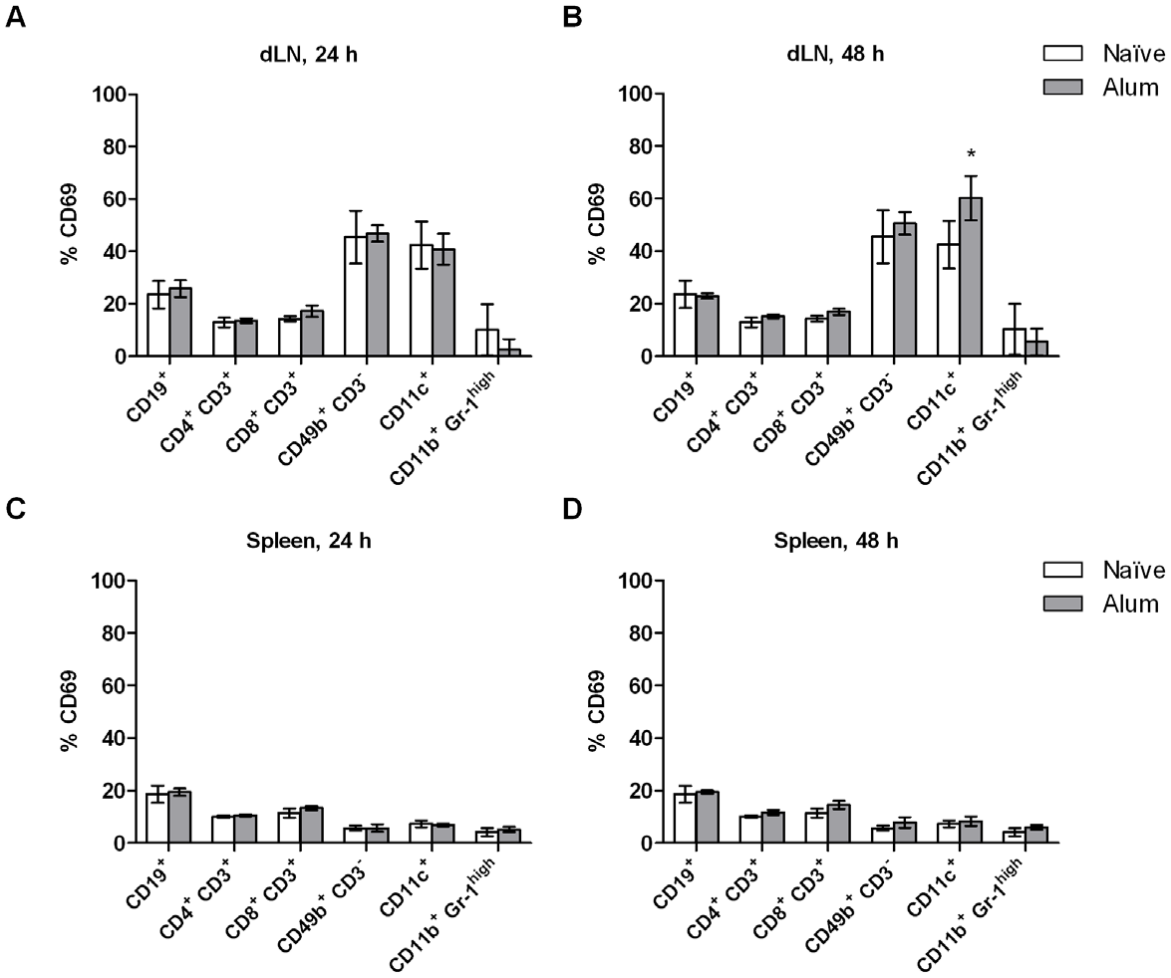
Expression of CD69 after Alum treatment. 1% Alum was injected subcutaneously at the base of the tail and at 24 or 48 h post treatment cells were prepared and the expression of CD69 on cells from the draining lymph nodes (dLNs) (A and B) or the spleen (C and D) were analyzed. Data is presented as mean ± SD (n = 3–6 mice). Significant differences between Alum treatment and naïve mice using Mann-Whitney test are outlined with *, *p<0.05*; **, *p<0.01* ; ***, *p<0.001*.

Early activation of cells in dLNs and spleen was monitored by analyzing expression of CD69. The expression of CD69 on B cells, T cell subsets, NK cells, DCs and granulocytes increased in dLNs at 24 and 48 h after Matrix-M™ treatment (Figure 6). Generally, at 24 h the 12 and 30 µg Matrix-M™ doses resulted in higher percentage of CD69^+^ cells compared to 3 µg Matrix-M™. However, at 48 h the CD69 expression in mice treated with 3 µg Matrix-M™ had increased to approximately the same level as with the higher doses evaluated. For all doses tested, splenocytes were not activated to the same degree as the lymph node cells and 3 µg Matrix-M™ did not significantly increase the percentage of CD69-expressing cell populations (Figure 6). Generally, the highest activation was detected at 48 h, CD4^+^ T cells and NK cells were activated by 12 and 30 µg Matrix-M™ and the percentage of CD69^+^ B cells and DCs increased after 12 µg Matrix-M™ treatment. Alum administration did not increase the percentage of CD69 expressing cells in dLNs or in spleen at 24 and 48 h with the exception of CD11c^+^ cells in dLNs at 48 h (Figure 7). Further, activation of B cells and DCs was studied by analyzing the expression of MHC class II and the co-stimulatory molecule CD86. In dLNs, 12 and 30 µg Matrix-M™ treatment resulted in increased levels of CD86 expression on B cells and DCs after 24 h (p<0.001, Table 1). The up-regulation of CD86 on B cells remained at 48 h while the expression on DCs returned to background level at this time point. In spleen the highest CD86 expression was seen after 48 h on both B cells and DCs (p<0.01 (12 µg) and p<0.001 (30 µg), Table 2). Up-regulation of MHC class II was mainly detected on DCs in dLNs. The levels were approximately doubled 24 h post treatment in mice treated with 12 or 30 µg Matrix-M™ (p<0.001) this was not true for 3 µg Matrix-M™. At 48 h post injection the level of MHC class II on DCs in dLNs had decreased below background level for all doses tested (p<0.05 (12 µg and 30 µg), no significant decrease in 3 µg Matrix-M™-treated mice). In Alum-treated mice, the only significant change of the analyzed activation markers was detected as an increased CD86 expression (p<0.05, Table 3) on DCs 48 h post treatment in dLNs. This was not observed for the Matrix-M™-treated mice at this time point.

**Table 1:** Median fluorescence intensity (MFI) of CD86 and MHC class II on CD19^+^ or CD11c^+^ cells in draining lymph nodes post Matrix-M™ treatment.

Lymph nodes		CD86 (×10^3^)		MHC class II (×10^3^)	
		24 h	48 h	24 h	48 h
CD19	PBS	0.8±0.05	0.8±0.1	42±5	47±4
	Matrix-M™ 3 µg	1.7±0.8	1.9±0.6**	41±4	49±3
	Matrix-M™ 12 µg	2.8±0.3***	1.8±0.4*	51±7*	44±4
	Matrix-M™ 30 µg	2.4±0.3***	2.2±0.5***	42±5	50±5
CD11c	PBS	7.9±2.7	12±6.9	103±35	124±49
	Matrix-M™ 3 µg	19±8.6	9.5±3.5	128±37	67±39
	Matrix-M™ 12 µg	54±13***	9.8±2.2	208±35***	56±21*
	Matrix-M™ 30 µg	42±11***	15±8.2	187±37***	60±30*

Mice were injected subcutaneously at the base of the tail and the draining lymph nodes were harvested and analyzed as described in Materials and Methods. Data is shown as mean ± SD (n = 6–8 mice). Significant differences from PBS treatment using Kruskal-Wallis test with Dunn's posttest are outlined with *, *p<0.05*; **, *p<0.01*; ***, *p<0.001*.

**Table 2:** Median fluorescence intensity (MFI) of CD86 and MHC class II on CD19^+^ or CD11c^+^ cells in spleen post Matrix-M™ treatment.

Spleen		CD86 (×10^3^)		MHC class II (×10^3^)	
		24 h	48 h	24 h	48 h
CD19	PBS	0.9±0.02	0.9±0.02	28±4	31±3
	Matrix-M™ 3 µg	0.9±0.03	1.1±0.06	28±3	31±3
	Matrix-M™ 12 µg	1.0±0.04**	1.2±0.04**	27±3	30±2
	Matrix-M™ 30 µg	0.9±0.03	1.3±0.1***	23±3*	33±4
CD11c	PBS	1.6±0.2	1.5±0.2	46±8	48±4
	Matrix-M™ 3 µg	1.7±0.3	1.7±0.3	47±7	44±5
	Matrix-M™ 12 µg	2.0±0.4	3.1±0.2**	47±5	40±2*
	Matrix-M™ 30 µg	2.4±0.5***	3.0±0.9***	49±6	41±6*

Mice were injected subcutaneously at the base of the tail and the spleen was harvested and analyzed as described in Materials and Methods. Data is shown as mean ± SD (n = 6–8 mice). Significant differences from PBS treatment using Kruskal-Wallis test with Dunn's posttest are outlined with *, *p<0.05*; **, *p<0.01*; ***, *p<0.001*.

**Table 3:** Median fluorescence intensity (MFI) of CD86 and MHC class II on CD19^+^ or CD11c^+^ cells in draining lymph nodes (dLNs) and spleen post Alum treatment.

		CD86 (×10^3^)		MHC class II (×10^3^)	
		24 h	48 h	24 h	48 h
CD19 dLNs	Naïve	0.8±0.1	0.8±0.1	40±8	40±8
	Alum	0.8±0.1	0.7±0.02	44±4	37±3
CD11c dLNs	Naïve	6.2±6.0	6.2±6.0	34±17	34±17
	Alum	9.2±2.5	18±9.0*	42±13	54±10
CD19 Spleen	Naïve	1.8±0.1	1.8±0.1	24±4	24±4
	Alum	1.9±0.1	1.9±0.05	22±2	23±2
CD11c Spleen	Naïve	2.4±0.2	2.4±0.2	37±0.4	37±0.4
	Alum	2.4±0.1	2.5±0.1	34±2	37±2

Mice were injected subcutaneously at the base of the tail and the draining lymph nodes and spleen was harvested and analyzed as described in Materials and Methods. Data is shown as mean ± SD (n = 3–6 mice). Significant differences from naïve mice using Mann-Whitney test are outlined with *, *p<0.05*; **, *p<0.01*; ***, *p<0.001*.

## Discussion

Matrix-M™ is a potent adjuvant and its immunomodulatory activities have been proven in a number of species, however, the mechanisms behind these properties are not yet fully understood [11], [14]. In this study, we show that Matrix-M™ treatment results in a local and transient immune stimulation with recruitment of lymphocytes, macrophages and granulocytes to dLNs and spleen. The number of cells in dLNs increased about 3-fold after 48 h in Matrix-M™-treated mice compared to PBS control. These results are in agreement with previous findings with a similar adjuvant where recruitment of lymphocytes to dLNs was reported in sheep after administration of ISCOMATRIX™ [15]. Further, Matrix-M™ administration also leads to activation of lymphocytes, DCs and granulocytes in dLNs as shown by increased expression of CD69. The biological impact of up-regulation of this early activation marker is not entirely elucidated however it reflects functionality of the immune cells [16]. Additionally, Matrix-M™ induces maturation of antigen presenting cells, detected by elevated levels of CD86 and MHC class II. CD86 is central for priming and activation of naïve T cells. The MHC class II expression was primarily affected on DCs in dLNs 24 h after Matrix-M™ treatment and the expression was down-regulated after 48 h. The down-regulation of MHC class II on DCs after 48 h may be due to lack of an antigen to process and present to T helper (Th) cells. Although Matrix-M™ administration increased the number of cells in spleen these were not at all as activated compared to cells in the dLNs. This could indicate that the splenocytes are primarily not activated by Matrix-M™ directly, contrary to the lymph node cells. The spleen cells are most likely activated secondarily by activated migrating cells as shown by the increased expression of late activation markers such as CD86. Alum, in contrast, did not induce recruitment or activation of studied immune cells in dLNs or spleen at the chosen time points, except for an increased expression of CD69 and CD86 on DCs in dLN after 48 h. On the contrary, in Matrix-M™-treated mice the up-regulation of CD86 occurred already at 24 h suggesting a different kinetics in immune cell activation. The Alum findings are in agreement with previously published data showing no increased CD69 expression on B-, T- or NK-cells as well as no increased CD86 expression on B cells or increased CD86/MHC class II expression on DCs at 24 h post injection [17]. The observed differences in early immune cell activation and recruitment between Matrix-M™ and Alum clearly implicate distinct adjuvant mechanisms of action.

The murine therapeutic dose interval of Matrix-M™ is between 3 and 12 µg. Three µg Matrix-M™ still recruited and activated immune cells, although the kinetics was to some extent delayed compared to higher doses tested. An administration of 12 µg Matrix-M™ induced a somewhat elevated serum level of IL-6 and the high-dose of Matrix-M™ induced a higher and a more heterogeneous secretion of IL-6 and also MIP-1β. Circulating levels of IL-6 and MIP-1β most likely represent spillover of locally produced cytokines and the secretion of these cytokines at the injection site may facilitate *e.g.* antigen presentation. IL-6 has been shown both *in vitro* and *in vivo* to be induced by a number of saponin-based adjuvants [18], [19]. IL-6 is primarily secreted by tissue-resident macrophages and provides signals for migration and activation of innate and adaptive immune cells. In particular, IL-6 is essential for induction of IL-21 production by naïve CD4^+^ T cells upon antigen stimulation. IL-21 promotes production of most IgG subclasses [20], [21]. MIP-1β, also produced by macrophages, is a potent chemoattractant for human granulocytes, NK cells and activated T cells. Previously, Alum and MF59 have been shown to induce MIP-1β *in vitro* [22]. However in this study, as for the 3 µg Matrix-M™ treatment, Alum treatment did not result in increased serum levels of the analyzed cytokines/chemokines.

The CD11b^+^ Gr-1^high^ granulocytes, predominantly neutrophils [23] was the population increasing the most in dLNs after Matrix-M™ treatment relative to the PBS control. This recruitment was detected even for the lowest dose evaluated nevertheless, the kinetics of the granulocyte migration was dose dependent. The highest number of cells was detected at the earliest time point analyzed, 4 h. Thus, it would be interesting to study the cell recruitment to the dLNs at an even earlier time point to further investigate the kinetics of granulocyte migration. The large recruitment of granulocytes to dLNs could influence the immune response in different ways. Several studies in mice show that neutrophils can transport live bacteria and antigen to dLNs [24]–[27]. This indicates a role for neutrophils in the adaptive immune response, possibly by delivering antigens to DCs [28]. It is also reported that CD11b on activated neutrophils interact with the c-type lectin DC-SIGN present on DCs leading to activation, providing an important link between innate and adaptive immunity [29]. These DCs can thereafter activate T cells and induce Th1 polarization. Interestingly, granulocytes with ingested microbes have shown to be involved in antigen presentation and to serve as substrate for *in vivo* cross-priming of CD8^+^ T cells by DCs [30], [31]. In addition, the adjuvant MF59 targets granulocytes, monocytes and macrophages, inducing an increased migration of these cells to the injection site [22]. However, it was shown that neutrophil depletion have no impact on the adjuvant effects of MF59, thus neutrophils and other cell populations possess overlapping functions [32]. Further, neutrophils have a negative impact on Th- and B-cell responses towards antigen formulated with *e.g.* Alum [33]. As Matrix-M™ also augments recruitment of neutrophils and DCs to the injection site, indicated by elevated cell numbers in dLNs, an increase in early antigen uptake and transportation may be achieved. In contrast, Alum treatment did not increase the neutrophil or DC numbers in dLNs nor in spleen.

The nanoparticle property of Matrix-M™ is likely essential for the ability to increase antigen uptake by phagocytosis, enabling antigen presentation. It has been shown that induction of innate immune responses by other particulate adjuvants *e.g.* aluminium-containing adjuvants is due to activation of the NLRP3 inflammasome [34]–[36]. However, if this activation is essential for the adjuvant effects on the adaptive immunity is yet a matter of debate [37], [38].

NK cells also increased in dLNs after Matrix-M™ treatment. Interestingly, it has been shown that DCs activated with *e.g.* LPS or CpG, activates and recruits NK cells to antigen-stimulated lymph nodes. Consequently, due to IFN-γ produced by NK cells there is a polarization towards a Th1 immune response [39], [40].

Taken together, the data presented show that low doses of Matrix-M™ induce a local transient proinflammatory response with recruitment, activation and maturation of important immune cells. Most likely the recruitment and activation of neutrophils and DCs by Matrix-M™ represents an important mechanism for the adjuvants' capacity to induce cytotoxic T lymphocytes. As an emerging body of evidence show that immune cells recruited after administration of several adjuvants seems to have overlapping functions, further investigations to decipher the mode of action of Matrix-M™ are required. In conclusion, we suggest that the described properties of Matrix-M™ may play a central role for efficient uptake and presentation of antigens, being significant for design and development of effective vaccines.

## References

[1] Leroux-Roels G (2010). Unmet needs in modern vaccinology: adjuvants to improve the immune response.. Vaccine.

[2] Dalsgaard K (1974). Saponin adjuvants. 3. Isolation of a substance from Quillaja saponaria Molina with adjuvant activity in food-and-mouth disease vaccines.. Archiv fur die gesamte Virusforschung.

[3] Cloete M, Dungu B, Van Staden LI, Ismail-Cassim N, Vosloo W (2008). Evaluation of different adjuvants for foot-and-mouth disease vaccine containing all the SAT serotypes.. The Onderstepoort journal of veterinary research.

[4] Morein B, Sundquist B, Hoglund S, Dalsgaard K, Osterhaus A (1984). Iscom, a novel structure for antigenic presentation of membrane proteins from enveloped viruses.. Nature.

[5] Lovgren K, Morein B (1988). The requirement of lipids for the formation of immunostimulating complexes (iscoms).. Biotechnology and applied biochemistry.

[6] Sjolander A, van't Land B, Lovgren Bengtsson K (1997). Iscoms containing purified Quillaja saponins upregulate both Th1-like and Th2-like immune responses.. Cellular immunology.

[7] Takahashi H, Takeshita T, Morein B, Putney S, Germain RN (1990). Induction of CD8+ cytotoxic T cells by immunization with purified HIV-1 envelope protein in ISCOMs.. Nature.

[8] Lovgren Bengtsson K, Morein B, Osterhaus AD (2011). ISCOM technology-based Matrix-M adjuvant: success in future vaccines relies on formulation.. Expert review of vaccines.

[9] Schnurr M, Orban M, Robson NC, Shin A, Braley H (2009). ISCOMATRIX adjuvant induces efficient cross-presentation of tumor antigen by dendritic cells via rapid cytosolic antigen delivery and processing via tripeptidyl peptidase II.. Journal of immunology.

[10] Drane D, Gittleson C, Boyle J, Maraskovsky E (2007). ISCOMATRIX adjuvant for prophylactic and therapeutic vaccines.. Expert review of vaccines.

[11] Madhun AS, Haaheim LR, Nilsen MV, Cox RJ (2009). Intramuscular Matrix-M-adjuvanted virosomal H5N1 vaccine induces high frequencies of multifunctional Th1 CD4+ cells and strong antibody responses in mice.. Vaccine.

[12] McKenzie A, Watt M, Gittleson C (2010). ISCOMATRIX() vaccines: Safety in human clinical studies.. Human vaccines 6.

[13] Heldens JG, Pouwels HG, Derks CG, Van de Zande SM, Hoeijmakers MJ (2010). Duration of immunity induced by an equine influenza and tetanus combination vaccine formulation adjuvanted with ISCOM-Matrix.. Vaccine.

[14] Radosevic K, Rodriguez A, Mintardjo R, Tax D, Bengtsson KL (2008). Antibody and T-cell responses to a virosomal adjuvanted H9N2 avian influenza vaccine: impact of distinct additional adjuvants.. Vaccine.

[15] Windon RG, Chaplin PJ, Beezum L, Coulter A, Cahill R (2000). Induction of lymphocyte recruitment in the absence of a detectable immune response.. Vaccine.

[16] Marzio R, Mauel J, Betz-Corradin S (1999). CD69 and regulation of the immune function.. Immunopharmacology and immunotoxicology.

[17] Caproni E, Tritto E, Cortese M, Muzzi A, Mosca F (2012). MF59 and Pam3CSK4 Boost Adaptive Responses to Influenza Subunit Vaccine through an IFN Type I-Independent Mechanism of Action.. Journal of immunology.

[18] Windon RG, Chaplin PJ, McWaters P, Tavarnesi M, Tzatzaris M (2001). Local immune responses to influenza antigen are synergistically enhanced by the adjuvant ISCOMATRIX.. Vaccine.

[19] Behboudi S, Morein B, Villacres-Eriksson M (1997). In vivo and in vitro induction of IL-6 by Quillaja saponaria molina triterpenoid formulations.. Cytokine.

[20] Dienz O, Eaton SM, Bond JP, Neveu W, Moquin D (2009). The induction of antibody production by IL-6 is indirectly mediated by IL-21 produced by CD4+ T cells.. The Journal of experimental medicine.

[21] Ozaki K, Spolski R, Feng CG, Qi CF, Cheng J (2002). A critical role for IL-21 in regulating immunoglobulin production.. Science.

[22] Seubert A, Monaci E, Pizza M, O'Hagan DT, Wack A (2008). The adjuvants aluminum hydroxide and MF59 induce monocyte and granulocyte chemoattractants and enhance monocyte differentiation toward dendritic cells.. Journal of immunology.

[23] Mohr E, Serre K, Manz RA, Cunningham AF, Khan M (2009). Dendritic cells and monocyte/macrophages that create the IL-6/APRIL-rich lymph node microenvironments where plasmablasts mature.. Journal of immunology.

[24] Maletto BA, Ropolo AS, Alignani DO, Liscovsky MV, Ranocchia RP (2006). Presence of neutrophil-bearing antigen in lymphoid organs of immune mice.. Blood.

[25] Chtanova T, Schaeffer M, Han SJ, van Dooren GG, Nollmann M (2008). Dynamics of neutrophil migration in lymph nodes during infection.. Immunity.

[26] Abadie V, Badell E, Douillard P, Ensergueix D, Leenen PJ (2005). Neutrophils rapidly migrate via lymphatics after Mycobacterium bovis BCG intradermal vaccination and shuttle live bacilli to the draining lymph nodes.. Blood.

[27] Bonneau M, Epardaud M, Payot F, Niborski V, Thoulouze MI (2006). Migratory monocytes and granulocytes are major lymphatic carriers of Salmonella from tissue to draining lymph node.. Journal of leukocyte biology.

[28] Megiovanni AM, Sanchez F, Robledo-Sarmiento M, Morel C, Gluckman JC (2006). Polymorphonuclear neutrophils deliver activation signals and antigenic molecules to dendritic cells: a new link between leukocytes upstream of T lymphocytes.. Journal of leukocyte biology.

[29] van Gisbergen KP, Sanchez-Hernandez M, Geijtenbeek TB, van Kooyk Y (2005). Neutrophils mediate immune modulation of dendritic cells through glycosylation-dependent interactions between Mac-1 and DC-SIGN.. The Journal of experimental medicine.

[30] Tvinnereim AR, Hamilton SE, Harty JT (2004). Neutrophil involvement in cross-priming CD8+ T cell responses to bacterial antigens.. Journal of immunology.

[31] Radsak M, Iking-Konert C, Stegmaier S, Andrassy K, Hansch GM (2000). Polymorphonuclear neutrophils as accessory cells for T-cell activation: major histocompatibility complex class II restricted antigen-dependent induction of T-cell proliferation.. Immunology.

[32] Calabro S, Tortoli M, Baudner BC, Pacitto A, Cortese M (2011). Vaccine adjuvants alum and MF59 induce rapid recruitment of neutrophils and monocytes that participate in antigen transport to draining lymph nodes.. Vaccine.

[33] Yang CW, Strong BS, Miller MJ, Unanue ER (2010). Neutrophils influence the level of antigen presentation during the immune response to protein antigens in adjuvants.. Journal of immunology.

[34] Hornung V, Bauernfeind F, Halle A, Samstad EO, Kono H (2008). Silica crystals and aluminum salts activate the NALP3 inflammasome through phagosomal destabilization.. Nature immunology.

[35] Eisenbarth SC, Colegio OR, O'Connor W, Sutterwala FS, Flavell RA (2008). Crucial role for the Nalp3 inflammasome in the immunostimulatory properties of aluminium adjuvants.. Nature.

[36] Kool M, Petrilli V, De Smedt T, Rolaz A, Hammad H (2008). Cutting edge: alum adjuvant stimulates inflammatory dendritic cells through activation of the NALP3 inflammasome.. Journal of immunology.

[37] Bauernfeind F, Ablasser A, Bartok E, Kim S, Schmid-Burgk J (2011). Inflammasomes: current understanding and open questions.. Cellular and molecular life sciences : CMLS.

[38] McKee AS, Munks MW, MacLeod MK, Fleenor CJ, Van Rooijen N (2009). Alum induces innate immune responses through macrophage and mast cell sensors, but these sensors are not required for alum to act as an adjuvant for specific immunity.. Journal of immunology.

[39] Zanoni I, Foti M, Ricciardi-Castagnoli P, Granucci F (2005). TLR-dependent activation stimuli associated with Th1 responses confer NK cell stimulatory capacity to mouse dendritic cells.. Journal of immunology.

[40] Martin-Fontecha A, Thomsen LL, Brett S, Gerard C, Lipp M (2004). Induced recruitment of NK cells to lymph nodes provides IFN-gamma for T(H)1 priming.. Nature immunology.

